# From Scarcity Cues and Social Proof to FoMO: A Dual-Mediation Model of Urgency in Consumer Decision-Making

**DOI:** 10.3390/bs16071250

**Published:** 2026-07-22

**Authors:** Severina Pal, Borut Milfelner

**Affiliations:** Faculty of Economics and Business, University of Maribor, 2000 Maribor, Slovenia; severina.pal@student.um.si

**Keywords:** fear of missing out (FoMO), scarcity effect, urgency effect, social proof, stimulus–organism–response (S-O-R) model, digital marketing, online consumer behavior, dual mediation

## Abstract

This research examines the psychological mechanisms that drive consumers’ fear of missing out (FoMO) in the context of digital marketing. Consumers are increasingly influenced by market dynamics that affect product availability and vendors’ supply chains, often alongside the widespread impact of suppliers’ marketing messages and social factors. Using the S-O-R (stimulus–organism–response) model, we test a double-mediation model via structural equation modeling on a sample of online customers. The main goal of the study is to examine how the effects of social proof and perceived scarcity create a sense of urgency, thereby influencing FoMO. The results indicate that customers’ perceptions of scarcity have a strong and statistically significant effect on urgency, while social proof exerts a weaker yet notable influence. Moreover, urgency significantly amplifies both the socially related component and the general fear of missed opportunities. Mediation analyses reveal that perceived scarcity affects FoMO indirectly through urgency. Although this supports the proposed mediation pathway, the substantial empirical overlap between perceived scarcity and urgency suggests that this relationship should be interpreted with caution. In contrast, social proof’s indirect effects are not statistically significant. These findings suggest that urgency serves as a crucial psychological mechanism mediating the influence of perceived scarcity on consumers’ emotional responses, while social proof may operate through alternative channels, such as social comparison or normative influence. This paper contributes to the S-O-R model and offers new insights into commodity theory and cognitive dissonance theory.

## 1. Introduction

From the moment users begin browsing the web and social networks, they are exposed to numerous pieces of information, as well as digital sales promotions and advertisements featuring attractive offers and exclusive deals. These promotions are designed to capture the customers’ attention and encourage them to make a purchase.

Advertisers often choose to use highly emotional messages in their advertising and sales promotions. These messages trigger strong emotional responses, increasing the likelihood that customers will remember them (Khuong & Tram, 2015). In addition to emotionally charged messages, advertisers employ other strategies to pique customers’ interest, often by presenting limited-time or limited-quantity offers. These tactics aim to instill a sense of urgency, encouraging consumers to take advantage of the offer as soon as possible. Many of these strategies are effective because they are rooted in psychological factors that influence consumer behavior. When a company informs customers that a product is available only in limited quantities or for a short period, it often prompts customers to explore the offer further, especially if they feel motivated to purchase. This is frequently tied to the fear of missing out (FoMO). Additionally, other individuals in a digital environment, such as friends, acquaintances, or fellow consumers, play an important role in shaping perceptions. While they may not directly promote products, their communications can be just as impactful—if not more so—than those of companies themselves.

This is particularly important in a time marked by frequent and intense global crises, including the COVID-19 pandemic and wars in Ukraine and the Middle East. These crises have a significant impact on vendors’ supply chains, product availability, and communication practices. As a result, psychological mechanisms such as FoMO, urgency, and perceived scarcity are greatly intensified. The combination of these crises and marketing communication efforts creates an environment that triggers FoMO, as consumers fear losing access to products. This is evident in issues such as chip shortages, rising prices, and food and energy shortages. Additionally, strong psychological pressure is generated by messages of urgency in marketing communications.

The importance of this research arises from the fact that consumers, particularly in digital markets, are constantly exposed to various marketing signals, such as limited inventory, limited-time offers, and popular products. Understanding how scarcity and social proof influence the fear of missing out (FoMO) through a sense of urgency is important for marketers. These factors can directly affect impulsive decisions and real-time buying behavior. The insight enables the development of more effective marketing communication strategies and more targeted campaigns. Additionally, it enables the optimization of web interfaces, and helps providers to better address consumers’ psychological mechanisms.

Many past studies have primarily examined the link between Fear of Missing Out (FoMO) and impulse buying (Good & Hyman, 2021; J. Zhang et al., 2021). These studies often also address post-purchase regret associated with reckless purchases (Saleh, 2026; Çelik et al., 2019). Additionally, research highlights that FoMO may arise from customers’ desires to follow trends and maintain social status (Fumar et al., 2023; Putri & Sa’id, 2024; X. Zhang & Rosli, 2025; Przybylski et al., 2013; Mahmud et al., 2023). This pressure often leads to impulsive purchases as customers seek to be part of the experience associated with popular products or to belong to groups already using them.

X. Zhang and Rosli (2025) have also connected FoMO with the scarcity effect. Authors who have previously explored the relationship between FoMO and a sense of urgency (Tiemessen et al., 2023; Oberoi, 2024; Zamfir, 2024) additionally speculate that there is a significant connection between the two constructs. Tiemessen et al. (2023) conclude that when clients experience a sense of urgency, it tends to increase their FoMO.

Gierl and Huettl (2010) demonstrated that the emergence of a positive scarcity effect depends on a product’s suitability for conspicuous consumption. Specifically, when a product is utilized for conspicuous consumption, indicators of scarcity resulting from limited supply are perceived as more advantageous than those arising from high demand. Additionally, Aral and Walker (2012) elucidate the role of social proof in shaping consumer preferences and influencing purchasing decisions. S. Gupta and Gentry (2019) provided empirical evidence that scarcity creates a sense of urgency among consumers, influencing their psychological responses. In contrast, other research has primarily focused on how communication strategies that highlight expiration can increase the urgency to make a quick purchase (Oberoi, 2024; Godinho et al., 2016). They emphasize that warning consumers about the limited availability of a desirable product can impact the entire decision-making process.

The available literature underscores the significant impact of stimuli, such as scarcity, social proof, and urgency, on consumer behavior, particularly in relation to urgency and the Fear of Missing Out (FoMO). These concepts are frequently examined in isolation or in limited combinations, leading to varied behavioral outcomes.

Furthermore, existing research often fails to clearly distinguish between scarcity, an objective indicator of limited availability, and urgency, a subjective sense of time pressure. While some studies suggest that scarcity can evoke a sense of urgency, empirical evidence supporting this relationship and its underlying psychological mechanisms is still limited. Distinguishing between these constructs is important because consumers may recognize scarcity without necessarily experiencing psychological urgency or FoMO. According to previous research, both constructs can independently influence FoMO (e.g., X. Zhang & Rosli, 2025; Tiemessen et al., 2023), but the effects companies aim to achieve in practice are often combined. This calls for an understanding of the combined effects and mediating influences, especially since it has been demonstrated that urgency—though influenced by various factors—can accelerate decision-making and reduce the amount of information processed (Dhar & Nowlis, 1999; Godinho et al., 2016), thereby intensifying feelings of FOMO.

The effects of social proof are rooted in social influence and conformity processes (Berger, 2013; Aral & Walker, 2012). Notably, there is a surprising paucity of studies that would systematically investigate the interplay between scarcity cues and social proof, particularly regarding their combined effects on urgency perceptions and the psychological pressure that leads to FoMO (Alfina et al., 2023; Zamfir, 2024). The relationship between scarcity and urgency may seem intuitive when products or opportunities are in limited supply. However, the mechanism through which social proof contributes to urgency is equally important but underexplored. In digital environments, signals indicating that many others are interested in or purchasing a product may intensify consumers’ perceptions that immediate action is necessary in order to avoid social exclusion, missed experiences, or loss of access to socially valued opportunities. Examining this relationship extends current understanding of urgency beyond traditional scarcity-based explanations and positions social proof as an additional driver of temporal pressure and FoMO in online consumer contexts.

Since these concepts are often examined individually or treated as straightforward linear predictors such approach lacks a deeper understanding of the psychological processes that transform these external influences into the subjective experience of FoMO. Combining scarcity and social proof originating from distinct theoretical foundations, this study proposes that both cues may influence FoMO through a shared psychological process characterized by perceived urgency. It views urgency not just as a statistical mediator, but as a key psychological mechanism that influences how individuals interpret and respond to external market signals. Therefore, urgency reflects the subjective experience of time pressure. It arises from the cognitive interpretation of two factors: limited availability (scarcity) and social information about a product’s popularity or acceptance (social proof). By identifying urgency as the mechanism through which external marketplace signals are transformed into internal psychological pressure, our research advances current understanding of how FoMO emerges in digital consumption environments.

We built our research on the Stimulus–Organism–Response (S-O-R) model, developing and empirically validating an integrated model. It enhances the understanding of the psychological processes involved in the digital shopping experience, particularly in an online environment. It makes an important contribution by introducing a dual mediation structure that outlines two parallel pathways: one examining how scarcity influences FoMO through a sense of urgency, and the other examining how social proof does so as well. By identifying urgency as a common mediating factor in both pathways, it allows for a direct comparison of the strength and significance of these impacts. This approach enhances previous studies by moving beyond simplistic, one-dimensional models and providing a deeper understanding of how various external stimuli affect FoMO through a shared psychological process.

## 2. Literature Review

### 2.1. Stimulus–Organism–Response (S-O-R) Model

This study is based on the Stimulus–Organism–Response (S-O-R) model, introduced by Albert Mehrabian and James A. Russell in 1974, in environmental psychology. The model was developed to explain how environmental factors influence individuals’ internal states and, in turn, shape their behavior. According to this, external stimuli from the environment trigger internal psychological processes within an individual, which then lead to behavioral responses. Internal processes may occur consciously or unconsciously as individuals interpret and evaluate environmental cues (Mehrabian & Russell, 1974; Hochreiter et al., 2023).

**Figure 1:**
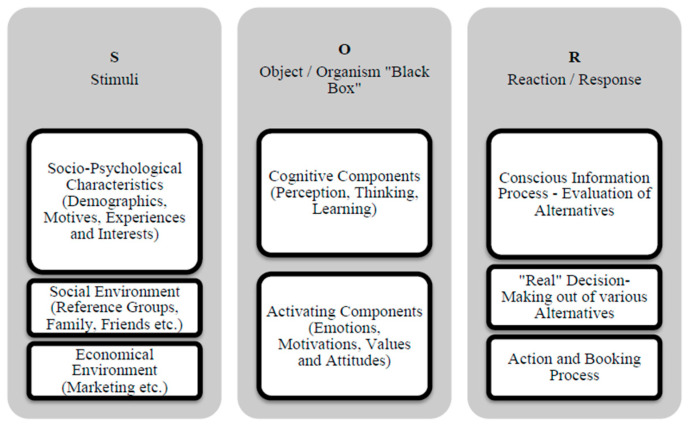
S-O-R model. Adapted from Hochreiter et al. (2023), based on Decrop (2006) and Freyer (2011).

The S-O-R model presented in Figure 1 conceptualizes behavior as a process consisting of three interconnected components: stimulus (S), organism (O), and response (R). Environmental stimuli are external signals that individuals encounter in their surroundings that can trigger internal evaluations and reactions. Stimuli are processed by the organism, i.e., the individual’s internal psychological processes. The processing of stimuli within the organism then leads to behavioral responses (Hochreiter et al., 2023).

The stimulus (S) represents external environmental cues or information that can influence an individual’s behavior. In consumer contexts, stimuli include marketing and social signals encountered in the environment, such as promotional offers, product advertisements, recommendations from other consumers, or information about trending products. In online retail environments, stimuli can be understood as the total set of cues presented through the website interface, including visual elements, information signals, and indicators of other users interacting with the platform (Eroglu et al., 2001). These cues can attract attention and elicit psychological responses in consumers.

The organism (O) represents the internal psychological processes through which individuals interpret and evaluate stimuli. These processes involve both cognitive and affective mechanisms. Cognitive processes include the perception, interpretation, and evaluation of information from the environment, while affective processes involve emotional reactions and psychological arousal, such as excitement, anxiety, or anticipation. In the context of online shopping environments, these internal states include both affective reactions, such as emotional responses and arousal, and cognitive evaluations, including beliefs, attitudes, and information processing (Eroglu et al., 2001). Through these internal processes, individuals assign meaning to external stimuli, which in turn influences how they respond.

The response (R) represents the behavioral outcomes that result from the processing of stimuli within the organism. In consumer behavior research, responses typically include observable behavioral intentions or actions, such as purchase intention, impulse buying, or brand or product engagement. These responses reflect how individuals react after interpreting and evaluating environmental cues.

An important assumption of the S-O-R model is that behavior cannot be explained solely by external stimuli. Instead, the model emphasizes the organism’s mediating role. Consumers interpret stimuli differently depending on their perceptions, motivations, emotions, and prior experiences, which explains why individuals may respond differently to the same environmental cue. As noted by Hochreiter et al. (2023), the S-O-R model provides a useful framework for understanding how consumers process information and make decisions in response to environmental stimuli. Consequently, the model has been widely applied to explain consumer behavior in online and digital marketing environments (Mehrabian & Russell, 1974; Eroglu et al., 2001; Kaur et al., 2017; Bothma & Van Staden, 2025).

In the context of this study, marketing cues such as scarcity signals and social proof represent environmental stimuli, while perceived urgency and fear of missing out represent internal psychological reactions within the organism stage.

### 2.2. The Fear of Missing Out

The theoretical foundation of FoMO is rooted in Self-Determination Theory (SDT). It proposes that human motivation is driven by three basic psychological needs: competence, autonomy, and relatedness (Ryan & Deci, 2000). When these needs are not adequately met, individuals become more susceptible to FoMO as they seek social interaction and validation by observing and participating in others’ experiences (Przybylski et al., 2013). In digital environments, individuals are often exposed to information about others’ activities and experiences through social media and other online platforms. This constant visibility of others’ experiences may intensify the need for social interaction and belonging, thereby increasing individuals’ susceptibility to experiencing FoMO. The effect is further reinforced by real-time content, promotional messages, and social interactions that continuously highlight others’ activities and consumption behaviors (Gaur et al., 2026).

The digital environment facilitates relationships between individuals with shared characteristics while simultaneously enabling continuous observation of others’ activities. According to Hayran et al. (2016), access to and exchange of information have become easier than ever, while exposure to others’ behavior has become unavoidable due to the widespread use of social media.

Through these platforms, consumers are constantly exposed to trends, popular content, and others’ activities, including purchases, events, experiences, and product reviews. Although this level of connectivity offers numerous advantages, it also introduces psychological pressures. Individuals who feel a strong need to remain informed about current trends and social activities may experience anxiety when they believe they are missing valuable information or experiences (Mahmud et al., 2023). This phenomenon is commonly referred to as fear of missing out (FoMO).

FoMO is defined as a pervasive fear that others are having rewarding experiences from which one is absent, accompanied by a strong desire to remain continuously connected to what others are doing (Przybylski et al., 2013). Individuals experiencing FoMO may feel concerned that others have more desirable experiences, opportunities, or products, which can create anxiety about missing out on valuable opportunities (Good & Hyman, 2021; Djamhari et al., 2024).

FoMO is therefore closely related to the individual’s desire to remain connected, included, and socially aligned with others (Hayran et al., 2020). People want to stay informed and connected to others’ experiences to prevent the feeling of being “left behind,” which threatens their self-concept (van der Schyff et al., 2022). by the desire to stay continually connected with what others are doing” (Przybylski et al., 2013). Importantly, FoMO does not necessarily arise from deliberate social exclusion. It can also occur when individuals become aware of experiences they are not involved in, even when exclusion was not intentional. This implies that FoMO is closely related to social comparison processes, where individuals evaluate their own experiences relative to those of others.

In digital environments, FoMO can significantly influence consumer behavior. Individuals experiencing FoMO are particularly sensitive to situations in which valuable opportunities may be missed. FOMO is often defined as the fear of missing out on a potentially lucrative opportunity. When consumers feel that a product or experience might become unavailable in the future, they often respond more strongly to marketing signals such as scarcity cues, limited-time promotions, or signs that other consumers place a high value on the same offering. As Neumann (2020) argues, the fear of missing out (FoMO) is rooted in uncertainty about potential negative future outcomes and is closely linked to anticipatory regret. Previous research suggests that FoMO can increase consumers’ impulsive purchasing tendencies, particularly when products are perceived as scarce or time-limited (Mahmud et al., 2023). This proves that FoMO heightens consumers’ sensitivity to potential opportunity loss and may amplify the effectiveness of marketing strategies that emphasize scarcity, urgency, and social proof.

Fear of Missing Out (FoMO) can be understood through the S-O-R model as an internal state of the individual (O) functioning as a psychological and emotional mechanism. External cues interact with unfulfilled basic needs, disrupting internal equilibrium. FoMO triggers various cognitive and emotional responses in consumers, such as anxiety and concern about missing out on opportunities, as well as a strong desire to stay socially connected. These internal feelings can prompt immediate purchase actions, including impulsive purchases, increased engagement with content, and heightened responsiveness to limited-time offers or popular products. Therefore, FoMO can also be viewed as an immediate psychological response (R).

### 2.3. The Urgency Effect

The effectiveness of urgency cues can also be explained by loss aversion, a concept derived from prospect theory developed by Kahneman and Tversky (1979). According to prospect theory, individuals evaluate outcomes relative to a reference point, perceiving them as gains or losses rather than as final wealth levels. Rather than evaluating all possible outcomes according to expected utility calculations, individuals tend to focus on potential gains and losses when making decisions. Importantly, losses are generally weighted more heavily than equivalent gains, meaning that individuals are typically more motivated to avoid potential losses than to pursue comparable gains.

The sense of urgency refers to a perceived need to initiate and complete an action immediately or soon. In consumer contexts, urgency is often deliberately heightened through marketing strategies that encourage immediate purchase decisions. According to Swain et al. (2006), urgency represents a psychological state in which individuals feel compelled to act quickly in response to a particular stimulus or opportunity. A sense of purchase urgency emerges when consumers feel compelled to buy a product immediately, thereby reducing their willingness to delay the decision (S. Gupta & Gentry, 2019).

One of the most common mechanisms for creating urgency in marketing is the use of time-limited offers, such as limited-time discounts, flash sales, coupons with expiration dates, and countdown promotions. These tactics signal that an offer will be available for only a limited time, encouraging consumers to act before the opportunity disappears. Such strategies create time pressure, defined as the perceived lack of sufficient time to evaluate available alternatives when deciding (Dhar & Nowlis, 1999; Godinho et al., 2016). Urgency cues can therefore influence consumer decision-making by increasing time pressure, reducing the extent of information processing, and encouraging faster purchase decisions. Decisions made under time pressure are associated with quicker responses and reduced deliberation. This suggests that urgency cues can prompt individuals to act quickly to capitalize on perceived opportunities, often leading to spontaneous or less carefully considered decisions. Under such conditions, consumers may evaluate fewer alternatives, rely more heavily on simple decision heuristics, and become more prone to impulsive purchasing behavior (Dhar & Nowlis, 1999; Lahoti, 2021).

In marketing contexts, time-limited offers may activate loss aversion because consumers can interpret the expiration of a promotion as a potential loss of an opportunity to obtain a favorable price or product. Consequently, consumers may act more quickly to avoid missing this opportunity, thereby accelerating decision-making in situations involving limited-time offers (Lahoti, 2021). In this way, urgency cues can increase consumers’ sensitivity to potential opportunity loss, thereby encouraging faster purchase decisions.

In the S-O-R framework, perceived urgency is an internal state of the Organism (O). It is influenced by external stimuli (S), such as a company’s communication about a time-limited offer or feelings of scarcity. However, the actual sensation of urgency is a psychological experience that occurs within the consumer. When individuals encounter time-sensitive cues, they process this information, leading to heightened psychological arousal and cognitive pressure. This can disrupt the consumer’s internal balance and create immediate psychological tension that seeks resolution through a behavioral Response (R).

### 2.4. The Scarcity Effect

One of the most relevant theoretical explanations of the scarcity effect comes from Commodity Theory, proposed by Brock (1968). According to this, the value of a good increases as its availability decreases. In other words, difficult-to-obtain objects, opportunities, or experiences are often perceived as more valuable. As a result, scarcity cues can increase the perceived value and desirability of products, thereby influencing consumer decision-making and increasing the likelihood of purchase.

Unlike urgency cues, which emphasize time limitations, scarcity cues highlight the limited availability of a product. It can be defined as an actual or perceived limitation in access to goods, services, or other resources that threatens consumers’ ability to satisfy their needs and desires (Hamilton et al., 2019). In addition, scarcity may activate the Scarcity heuristic, whereby individuals infer that scarce products are more valuable or desirable simply because they are difficult to obtain (Zamfir, 2024). When a product is perceived as scarce, consumers may attribute greater value to it simply because it is less available. This phenomenon suggests that limited availability can increase a product’s perceived desirability and value (Zamfir, 2024). Scarcity can also signal product quality and popularity, as consumers may assume that scarce products are preferred or of higher quality (Lynn, 1991; Van Herpen et al., 2009).

Scarcity cues may also enhance perceptions of exclusivity and social status. Owning scarce products can signal uniqueness or prestige, thereby increasing their appeal to consumers who value exclusivity (Gierl & Huettl, 2010; Roy & Sharma, 2015). As a result, scarcity can influence consumer evaluations of products and increase the likelihood of purchase.

Scarcity is commonly used in marketing communication through messages that emphasize the limited availability of products. Expressions such as “limited edition,” “available while supplies last,” or “only a few items left in stock” signal that a product is scarce and may soon become unavailable. In such situations, consumers may feel encouraged to act quickly to obtain the product before it becomes inaccessible.

In the S-O-R model, scarcity refers to environmental stimuli (S) that influence consumers’ urge to make immediate purchases. Scarcity stimuli can arise from objective circumstances, such as a limited supply of a product, or from a company’s marketing efforts that communicate a potential product scarcity. Additionally, exclusivity can accompany scarcity for certain products when supported by specific marketing strategies. Exclusivity serves as a stimulus that a product is rare, premium, limited to a select group, or not widely accessible. This exclusivity acts as an environmental cue, shaping consumers’ perceptions and emotional responses.

### 2.5. Social Proof

Social proof is embedded in comparison theory that proposes that individuals evaluate their own attitudes, behaviours, and preferences by comparing themselves with others (Festinger, 1954). In consumer contexts, social comparison occurs when individuals observe others’ consumption choices, such as which products people purchase, which brands are popular, or which trends others follow. These observations can influence consumers’ perceptions of product desirability and value. Research suggests that when consumers see others enjoying or using a particular product, their own interest in that product may increase (Ilyas et al., 2022, cited in Kopřivová & Bauerová, 2024). Similarly, products that appear popular or widely purchased may be perceived as more valuable and desirable (Zamfir, 2024).

Social comparison is closely related to the concept of social belonging. Individuals often adjust their behaviour to align with perceived social norms and gain acceptance within their social groups. This tendency is particularly evident among younger consumers, who frequently seek approval and a sense of belonging within their peer groups (Crosnoe, 2011; Eccles & Roeser, 2011; Park & McCallister, 2023). In some situations, conforming to social norms may involve purchasing specific products—such as clothing or other visible consumer goods—that signal membership in a particular social group.

Consumers rarely make purchase decisions entirely independently, as the opinions, experiences, and behavior of others often influence their choices. When individuals face uncertainty or lack sufficient information about a product, they frequently rely on cues from other consumers to guide their decisions. This phenomenon is known as social proof, a concept introduced by Cialdini (1984). Social proof refers to the tendency for individuals to adopt the behavior or decisions of others to determine what is appropriate or desirable in a given situation (Park & McCallister, 2023). In consumer contexts, social proof functions as an informational shortcut that helps individuals reduce uncertainty when evaluating products or services. When consumers observe that others have purchased, recommended, or positively evaluated a product, they may interpret this behavior as evidence that the product is trustworthy or valuable. As a result, the behavior and opinions of other consumers can significantly influence purchase decisions.

In digital environments, social proof is particularly visible through mechanisms such as online reviews, ratings, recommendations, and influencer endorsements. Modern technology allows consumers to easily access the experiences and opinions of other users through social media platforms and e-commerce websites. These signals help consumers reduce uncertainty and perceived risk when evaluating products in digital environments (Chen et al., 2022; Park & McCallister, 2023). Consequently, when consumers perceive that a product is widely used or well evaluated by others, they are more likely to consider it desirable and worth purchasing.

In digital environments, social influence is further reinforced through signals of social validation. Social validation refers to individuals’ tendency to seek approval and affirmation from others regarding their decisions and behavior, particularly in socially visible contexts (Ballara, 2023). On social media platforms and e-commerce websites, cues such as likes, shares, endorsements, and visible purchase activity provide signals that others approve of or support a particular product. These validation signals may strengthen consumers’ perceptions that a product is desirable or socially accepted, thereby influencing their consumption choices (Wellons et al., 2024).

In the context of the S-O-R theory, social proof acts as an environmental Stimulus (S). It signals to individuals that others are already making purchases, which the organism (O) then processes internally. In the digital environment, social proof appears through various cues, such as others’ shopping behavior, online product reviews, product views, total review counts, product sales, items saved in wish lists, and product endorsements. Social proof is often conveyed through external informational cues embedded in the digital landscape.

## 3. Conceptual Model and Hypothesis

Scarcity and urgency both limit the availability of an opportunity, but in different ways. Urgency restricts the time available to obtain an offer, whereas scarcity refers to the limited quantity of products available for purchase. Many researchers have suggested that scarcity cues can create a sense of urgency in consumers by signaling that an opportunity to obtain a product may soon disappear (S. Gupta & Gentry, 2019). Scarcity inherently reflects restricted availability, which may arise from limitations in quantity or time, thereby increasing the perceived risk that a product will become unavailable (Barton et al., 2022). In such situations, consumers may feel compelled to make decisions more quickly, reducing their willingness to delay the purchase (Gabler & Reynolds, 2013). This effect is particularly pronounced under perceived time pressure, when consumers simplify decision processes and reduce information search (Dhar & Nowlis, 1999; Godinho et al., 2016). When consumers perceive that a product is available in limited quantities or for a limited time, they may feel psychological pressure to act quickly to secure it before it becomes unavailable (S. Gupta & Gentry, 2019). Scarcity in marketing communication can be conveyed through different mechanisms. S. Gupta and Gentry (2019), building on Cialdini’s (2008) work, distinguish between limited-time scarcity and limited-quantity scarcity. Limited-time scarcity refers to situations in which products or promotions are available only for a specific period, whereas limited-quantity scarcity indicates that only a limited number of items remain available. In both cases, consumers may perceive a risk that the opportunity to obtain the product will disappear.

Scarcity signals such as countdown timers, messages indicating limited stock, or statements emphasizing the limited availability of an offer can increase perceived time pressure and encourage consumers to make faster decisions (Godinho et al., 2016; Zamfir, 2024). As a result, the perception of scarcity may strengthen consumers’ sense of urgency during the purchase decision process. Hence, we propose:

**H1.**  *The scarcity effect has a positive impact on the urgency effect.*

Social proof can influence consumer behavior by signaling that a product or offer is popular among other consumers. When individuals observe that many others are purchasing or recommending a product, they may interpret this behavior as an indication that the product is desirable or valuable. Research shows that observing others using or enjoying a product can increase consumers’ interest in that product (Kopřivová & Bauerová, 2024). Similarly, products that are perceived as popular or in high demand are often evaluated as more valuable and desirable (Zamfir, 2024). Importantly, the effect of social proof may be intensified when combined with scarcity cues. When consumers observe that many others are purchasing a product that is also in limited supply (e.g., “only a few items left” or “bestseller”), social proof provides an additional signal that the product is both desirable and at risk of becoming unavailable (Zamfir, 2024). This combination can increase perceived competition and reduce the perceived time available to act, thereby strengthening consumers’ sense of urgency. In digital environments, social proof is often reinforced through signals of social validation, such as likes, shares, endorsements, or visible purchase activity. These cues provide consumers with social approval and may strengthen the perception that obtaining the product is desirable or socially valued, thereby increasing the motivation to act quickly (Wellons et al., 2024).

Social proof can also activate consumers’ desire to conform to social norms and to belong to a particular group. Individuals often adjust their behavior to align with others’ actions and preferences, particularly when products are associated with social identity or group belonging (Crosnoe, 2011; Eccles & Roeser, 2011; Park & McCallister, 2023). When consumers perceive that a product is widely adopted or trending, they may feel pressure to obtain it quickly to keep up with the group or avoid being left out. Consequently, signals indicating that many others are purchasing or recommending a product may strengthen consumers’ sense of urgency during the purchase decision process. Therefore, we propose the following hypothesis:

**H2.**  *Social proof has a positive impact on the urgency effect.*

Consumers may experience fear of missing out when they perceive that an attractive opportunity could disappear if they do not act quickly. In marketing contexts, urgency cues are frequently used to signal that a product, discount, or promotional offer is available only for a limited time. Such signals can create a perception that delaying a decision may result in losing access to a valuable opportunity. Previous research suggests that urgency cues can intensify consumers’ fear of missing out on attractive opportunities. In digital environments characterized by real-time interactions and time-sensitive offers, urgency signals may amplify FoMO, prompting consumers to make faster purchase decisions to avoid missing a perceived opportunity (X. Zhang & Rosli, 2025). Time-limited offers—such as promotions available only for a short period—can create the perception that an opportunity may soon disappear. As a result, consumers may feel psychological pressure to make quick decisions to avoid missing a potentially valuable offer (Zamfir, 2024). Marketing messages that emphasize urgency, such as statements indicating that an offer is valid only for a limited time, are designed to encourage immediate action. These signals communicate that the opportunity to obtain a product or benefit is temporary, which may increase consumers’ concern that postponing the decision could result in losing access to the offer. When consumers already show interest in a product, urgency cues may further intensify this concern by highlighting the risk of missing the opportunity if they do not act quickly.

In digital environments, urgency cues are often reinforced through mechanisms such as countdown timers that visually indicate the limited time available to take advantage of a promotion. These signals can increase perceived time pressure and strengthen consumers’ fear that the opportunity may disappear if they fail to act promptly (Tiemessen et al., 2023; Oberoi, 2024). Consequently, we hypothesize the following:

**H3.**  *The urgency effect has a positive impact on the fear of missing out.*

When products are scarce—whether due to limited production or high demand—the perceived likelihood that the product may soon become unavailable can intensify consumers’ concerns about missing out. When availability is restricted, consumers may feel compelled to act quickly to secure the product before it sells out. Prior research suggests that scarcity cues can trigger psychological responses related to fear of missing out, as consumers become increasingly concerned that delaying their decision may result in losing access to the product (X. Zhang & Rosli, 2025). The likelihood of experiencing fear of missing out may be particularly strong when consumers perceive a product as highly desirable or valuable. In such situations, limited availability can create psychological pressure to act before the opportunity disappears. Sales promotions characterized by time restrictions and exclusivity can further amplify FoMO, as consumers perceive a limited opportunity to obtain a desired product (Djamhari et al., 2024).

This effect is consistent with commodity theory, which suggests that limited availability increases perceived value and desirability (Brock, 1968; Lynn, 1991). In this context, urgency can play an important mediating role. While scarcity conveys limited availability, urgency conveys the perceived need to act immediately. When scarcity cues are accompanied by time pressure, consumers experience heightened urgency, which intensifies psychological responses such as anticipated regret and anxiety about missing an opportunity (Swain et al., 2006; Good & Hyman, 2021). These emotional responses reinforce fear of missing out (FoMO), as individuals perceive that delaying action may result in losing access to a valued opportunity. Scarcity-induced urgency can further shift consumers’ focus to the risk of missing out on a valuable opportunity, often leading to faster, less reflective decision-making (Zamfir, 2024). This heightened sensitivity to potential loss amplifies FoMO, as consumers become increasingly motivated to act immediately to avoid missing the opportunity.

It is important to clarify that we do not suggest that FoMO is solely dependent on perceived urgency. FoMO can arise from various factors, and consumers may experience it even when no explicit urgency cues are present. However, we propose that urgency can significantly intensify FoMO in online shopping environments. Specifically, urgency functions as a cognitive-emotional amplifier, increasing the perceived risk of missing an opportunity and reducing the time available for deliberate decision-making. In this sense, urgency should not be viewed as a necessary precursor to FoMO, but rather as a situational factor that can explain how the impact on FoMO is built, particularly in combination with other cues such as scarcity.

The source of scarcity can vary across contexts, including supply restrictions, excess consumer demand, and time limitations (Barton et al., 2022). These different forms of scarcity convey distinct signals to consumers. For instance, supply-based scarcity emphasizes exclusivity and uniqueness, while demand-based scarcity signals popularity, and time-based scarcity primarily induces urgency. As a result, different types of scarcity generate varying levels of perceived urgency and psychological pressure. Limited editions or short-term offers give the impression that they are irreversible, while shortages due to high demand create a different sense of urgency, as customers expect products to be available again, but may also feel that the offer will be less favorable (e.g., a higher price). Such situations can cause varying degrees of urgency impact. While scarcity conveys information about availability, urgency reflects a more subjective assessment of the need for immediate action, which can prompt anticipation of regret and FoMO. Under time-based scarcity, the availability of an offer is restricted to a specific time frame, meaning that the offer itself, rather than the product, becomes the scarce resource (Barton et al., 2022; Aggarwal et al., 2011). This limitation creates a sense of urgency, as consumers must act before the opportunity expires. As a result, individuals may experience anticipated regret associated with missing out on the offer (Abendroth & Diehl, 2006), which intensifies emotional responses and reinforces FoMO. Consequently, time-based scarcity not only signals limited availability but also activates psychological pressure to act immediately.

On this basis, the urgency effect can be understood as a mediator between the scarcity effect and FoMO, meaning that scarcity increases perceived urgency, which in turn reinforces the fear of missed opportunity, and we put forward the following hypothesis:

**H4.**  *The urgency effect is a mediator between the scarcity effect and the fear of missing out.*

Fear of missing out is closely related to social comparison processes, in which individuals evaluate their own experiences and opportunities relative to those of others (Przybylski et al., 2013). When consumers observe the activities, preferences, or consumption choices of others, they may become concerned that they are missing out on valuable experiences or opportunities. In digital environments, this process is particularly evident on social media platforms, where users are frequently exposed to others’ activities and consumption behavior. Research has shown that posts by other users sharing their preferences and experiences can intensify fear of missing out (Hayran et al., 2016). Similarly, exposure to influencers and popular trends may heighten consumers’ concerns about missing out on desirable products or experiences promoted in their social networks (Mahmud et al., 2023). Social proof signals may therefore increase consumers’ motivation to act quickly to participate in the same experiences or obtain the same products as others. In such situations, social influence can intensify their sense of urgency, thereby intensifying their fear of missing out on perceived opportunities.

Social proof promotes an urgency effect, especially in environments where trends are rapidly created and changing. In such situations, others’ behavior serves not only as information about popularity but also as a signal for immediate action if an individual wants to remain part of current events (Berger, 2013; Aral & Walker, 2012). In doing so, the urgency effect acts as a mediator between social proof and FoMO. Social proof, in itself, does not necessarily cause fear of missing an opportunity; rather, it increases the perceived time constraint of the decision. When a person sees that others quickly make a specific choice, they interpret it as a sign that the opportunity is fleeting, which creates a sense of urgency. It is this perceived urgency that activates the emotional response characteristic of FoMO. Without it, social proof would have remained primarily an indicative signal of popularity rather than a trigger for the fear of delay. Therefore, the urgency effect is a key mechanism that translates the impact of social proof into FoMO. Urgency also transforms social information into perceived temporal pressure and emotional response.

While social proof provides cues about others’ behavior, it does not inherently require immediate action; urgency is what converts this information into a compelling need to act. When individuals observe that others are rapidly engaging in a behavior (especially on social networks), they interpret it as a sign of fleeting opportunity, which increases perceived time pressure and shifts decision-making from deliberative to more affect-driven processes. At the same time, urgency amplifies anticipated regret (Roese & Summerville, 2005), as inaction becomes associated with the risk of missing out, and reinforces perceived competitiveness for limited opportunities. Through these mechanisms, urgency translates social proof into a psychologically salient and emotionally charged FoMO response, thereby serving as its mediator. Therefore, urgency may mediate the relationship between social proof and fear of missing out, hence:

**H5.**  *The urgency effect is a mediator between social proof and the fear of missing out.*

**Figure 2:**
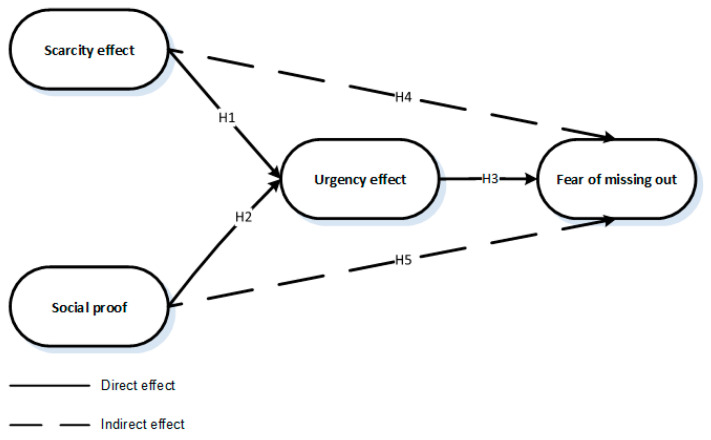
Conceptual framework and hypotheses.

Conceptual framework and hypotheses are presented in Figure 2.

## 4. Materials and Methods

### 4.1. Measurement Scales

The measurement instrument was created using a combination of items from existing literature and original items. The development process consisted of two phases. First, we assessed content validity with the help of four academics: two specialized in consumer behavior and two in marketing research. Following this, we developed a questionnaire in English, which was then translated into Slovenian using the back-translation procedure recommended by Harkness et al. (2010).

Fear of missing out (FOMO) was defined by Przybylski et al. (2013) as a general fear that others are having useful experiences in which the individual is not involved, and as a constant desire to maintain a relationship with others’ activities. To measure the construct, we adapted Przybylski et al.’s (2013) 8-item scale.

Two types of scarcity used in marketing communications are most often discussed in the literature: (a) shortages due to increased demand, and (b) shortages due to limited edition (Verhallen, 1982; Verhallen & Robben, 1994; Gierl & Huettl, 2010; Eisend, 2008; Roy & Sharma, 2015). On this basis, we have created a modified measurement scale that combines the findings of the aforementioned research and covers both types of scarcity while remaining the scale parsimonious. We measured the scarcity effect (SE) with five items.

To measure the urgency effect, we examined how advertisers encourage instant buying behavior, such as time-limited discounts, quick sales, countdown timers, and time-sensitive notifications (Oberoi, 2024; Tiemessen et al., 2023). On this basis, we have formulated four items that measure the urgency effect (UE).

We have operationalized social proof as a psychological factor based on observing and imitating others’ behavior. In line with the existing literature, we included ratings, opinions, and recommendations, as well as influencers’ influence on customer decisions. The scale is based on the findings of several studies (Lackermair et al., 2013; Chen et al., 2022; Arunkumar et al., 2023; Park & McCallister, 2023; Zamfir, 2024) and was designed as a five-item scale.

Respondents rated the level of agreement in all scales on a 7-point Likert scale.

### 4.2. Data Collection and Sample Characteristics

Data collection was conducted using an online questionnaire published on the 1KA Arnes platform. The survey link was available to all potential participants who received the URL. The survey questionnaire was disseminated via social media. We published a post on social media (Facebook and Instagram), outlining the purpose of the research and providing a link to the online questionnaire. This approach facilitated direct contact with a relevant group of respondents, thereby enhancing the survey’s visibility and improving response rates. While a convenience sample limits data representativeness, it still offers valuable insights into populations where random sampling is more challenging to implement.

Participants were asked to complete the questionnaire in the scenario of an online shopping context. Specifically, they were instructed to imagine browsing and purchasing products through digital platforms, where they could encounter typical online marketing stimuli such as time-limited offers, low-stock notifications, and indicators of other users’ activity. This study focused on consumer responses in digital environments, where urgency, scarcity, social proof, and fear of missing out are continuously embedded in real-time interactions and platform-based communication. Although the study has methodological limitations in terms of representativeness, the results offer important theoretical and practical insights that future research can validate with more diverse and representative samples.

A total of 217 individuals opened the survey, of whom 121 completed it in full, yielding a final sample and a 55.8% response rate. The achieved sample size meets the pre-set goal (between 100 and 150 participants). The average time to complete the survey was 3 min and 39 s, indicating a relatively short, time-saving survey tool. Sample characteristics are presented in Table 1.

Since the items used to construct exogenous and endogenous variables were obtained from the same informant group, we acknowledged the possibility of common-method bias. Therefore, Harman’s one-factor test was implemented, and showed that the first factor explained 46.86% of the variance of all items, which is less than the suggested 50%.

The sample consisted of 121 participants, of which 60.3% were women, 38.8% were men, and 0.8% were others. The majority of respondents were between 25 and 34 years old (46.3%), followed by those aged 18 to 24 (24%). In terms of education, secondary education (35.5%) was the most common, followed by university (26.4%) and tertiary education (20.7%), while a smaller share was represented by respondents with a master’s degree (10.7%) and a doctorate (1.7%).

Although the sample size (*N* = 121) was small for the covariance-based structural equation modeling method (CB-SEM), the literature suggests that acceptable sample adequacy depends not only on sample size. Also, research goals, model complexity, indicator reliability, communalities, and data characteristics should be considered (Kline, 2023). Therefore, the CB-SEM method was chosen, since partial least square SEM, which is usually more appropriate with small samples, should typically be applied in situations when the number of indicators is fewer than three, the distribution of variables is not normal, or when the research objective focuses on prediction and theory development, not on hypothesis testing (Reinartz et al., 2009).

Regarding the sample size, a post hoc statistical power analysis was also conducted. With a medium effect size (f^2^ = 0.15), α = 0.05, and four predictors in the structural model, the achieved sample size (*N* = 121) yielded statistical power of 0.995, which is above the recommended value of 0.80 (Cohen, 1988; Faul et al., 2009). This indicates adequate power for detecting medium-sized effects. Given the parsimonious structure of the proposed mediation model and the satisfactory measurement properties observed in the analysis, the sample size was deemed adequate for model estimation.

### 4.3. Dimensionality, Validity, and Reliability of the Scales

The dimensionality of the constructs was evaluated using confirmatory factor analysis within a CB-SEM framework in AMOS 28, with maximum likelihood estimation. In the process, we excluded two items on FoMO, two on the scarcity effect (SE), one on the urgency effect (UE), and one on social proof (SP). The items were excluded based on model fit indices and modification indices. The empirical evidence suggested that the FoMO construct could be better explained by two subconstructs, namely social-driven FoMO (SD-FoMO) and missed-opportunities FoMO (MO-FoMO). To assess whether a two-subconstruct structure was more suitable than a single construct structure, we compared two measurement models (a one-factor model vs. a two-factor model). Dimensionality was assessed by comparing the fit indices for both models. χ^2^ was equal to 33.11 at 8 df for the two-factor model, and 99.71 at 9 df for the single-factor model. A significant difference between the two models at *p* < 0.01 suggests that the multifactorial model is better suited to explain FOMO. A significant improvement in other fit indices also confirmed that result. It has to be noted that the two-factor structure of FoMO was not specified a priori as a formal measurement hypothesis but emerged during the measurement model evaluation process through confirmatory factor analysis. Given the growing conceptual distinction between socially driven concerns (Przybylski et al., 2013; Hayran et al., 2016; van der Schyff et al., 2022) and concerns about missed opportunities (Djamhari et al., 2024; Neumann, 2020; Mahmud et al., 2023) in recent FoMO literature, we considered this alternative structure theoretically plausible. Despite this, the two-factor solution should be interpreted as an exploratory refinement that requires further validation in future studies.

The overall measurement model fit was assessed with confirmatory factor analysis using several indicators. The results show an acceptable fit of the model to the data, despite the significant χ^2^ value (χ^2^(94) = 178.801). Since χ^2^ statistics are highly sensitive to sample size (Kaplan, 2000), we assessed other fit indices. TLI = 0.920, CFI = 0.937, and IFI = 0.938 exceed the limit of 0.90 recommended by Byrne (1994), Hu and Bentler (1999), and MacCallum et al. (1996). The RMSEA (0.087) just slightly exceeds the ideal limit of 0.08. his index is known to be sensitive to model complexity and may increase with the number of constructs included in the model (Breivik & Olsson, 2001). Given that the present model included only four constructs and that the remaining fit indices demonstrated appropriate model fit, the obtained RMSEA value can still be considered acceptable (MacCallum et al., 1996).

Next, we analyzed the reliability, convergent, and discriminant validity of SD-FOMO, MO-FOMO, SE, UE, and SP scales. The reliability of the constructs was evaluated using the composite reliability (CR) coefficient. The CR values for all constructs exceed the recommended limit of 0.7, indicating good internal consistency of the measuring scales. CR values range from 0.839 to 0.882, confirming the high reliability of all constructs (see Table 2).

Convergent validity was estimated based on the average variance extracted (AVE) and factor loadings (λ). All AVE values exceed the 0.5 threshold (ranging from 0.630 to 0.723), indicating that the constructs explain more than half of the variance in their indicators. Factors are high in most cases (λ > 0.7), which further confirms the corresponding convergent validity.

The results show that the measurement model has good reliability and adequate convergent validity. Discriminant validity was assessed using the Fornell–Larcker (Fornell & Larcker, 1981) criterion and the HTMT test, following the procedure proposed by Henseler et al. (2015). The results of the Fornell–Larcker test in Table 3 show that the square roots of AVE for all constructs (from 0.794 to 0.850) are higher than their correlations with each other, confirming the corresponding discriminant validity. Additional discriminant validity testing using the HTMT criterion indicates that all HTMT values, except one, are below the 0.85 threshold (see Table 4). The exception is the UE-SE pair (0.908), which exceeds the recommended limit, suggesting a potential substantive overlap between the constructs of perceived supply constraint and time urgency.

## 5. Results

The hypotheses were tested with a structural model estimated by maximum likelihood. The results indicate statistically significant direct and indirect effects among the considered constructs. Model fit indices indicate that the model fits the data well. The value of χ^2^ = 233.97 at df = 132 is statistically significant, which is usually the case for smaller samples. That is why other fit indices, which are less sensitive to smaller samples, were considered. Those incremental fit indices indicate a good fit of the model: TLI = 0.904, CFI = 0.926, and IFI = 0.928, which exceed the recommended value of 0.90 and represent an improvement over the baseline model. RMSEA = 0.080, which is exactly the recommended threshold and indicates a marginal or moderate deviation of the model from the data. Overall, the results show that the model has an adequate fit.

The direct impacts were assessed using the standardized regression weights. The indirect effects and their significance were assessed using bootstrapping, following the procedure outlined by Preacher and Hayes (2008). Confidence intervals and standard errors are reported in Table 5.

The results regarding the impact of latent variables show that SE has a strong, statistically significant effect on the UE (β = 0.787; *p* < 0.001), indicating that perceived scarcity significantly increases the sense of urgency. The H1 hypothesis was therefore confirmed.

SP significantly impacts the UE (β = 0.182; *p* < 0.05), indicating that social proof enhances the sense of urgency, albeit to a lesser extent than the scarcity effect, thereby confirming H2.

Furthermore, the results show that the urgency effect (UE) has a strong and statistically significant effect on both SD-FOMO (β = 0.557; *p* < 0.001) and MO-FOMO (β = 0.795; *p* < 0.001). This means that a sense of urgency significantly increases both dimensions of FOMO, thereby confirming H3.

As shown in Table 5, results indicate that SE has a statistically significant indirect effect on SD-FOMO (β = 0.438; *p* < 0.001) and on MO-FOMO (β = 0.625; *p* < 0.001). This suggests that SE affects FOMO primarily through a sense of urgency. The H4 hypothesis was thus confirmed.

On the other hand, the indirect effects of SP on SD-FOMO (β = 0.101; n.s.) and on MO-FOMO (β = 0.145; n.s.) are not statistically significant. This means that the urgency effect does not mediate the relationship between social proof and the fear of missing out in this model. Therefore, the H5 hypothesis was rejected.

Gender, age, and education were used to control the impact of demographics on urgency and FoMO. Results in Table 5 indicate that those variables did not have a significant impact on exogenous variables.

## 6. Discussion

Our research confirms that in the context of online shopping, psychological factors such as social proof, the scarcity effect, the urgency effect, and the FoMO do not operate independently. Instead, they are interconnected and often work together, combining concepts that have rarely been considered simultaneously in previous scientific literature.

The strongest relationship was observed between scarcity and urgency, meaning that when consumers perceive a product as having limited availability—whether in terms of time or quantity—they experience increased pressure to act quickly and make a purchase. This suggests that limited supply can create a sense of urgency, leading consumers to make impulsive and often unplanned purchases. This supports prior research, such as that of S. Gupta and Gentry (2019), who note that scarcity induces psychological urgency in consumers. Additionally, Cialdini (2008) explains that companies can communicate the effects of scarcity in two main ways: through time-limited supply or by offering a limited quantity of products. In both cases, this strategy encourages quicker, less deliberative purchasing decisions.

Building on the role of scarcity in generating urgency, we next interpret the impact of social proof. Social proof had a positive direct impact on feelings of urgency. Our research confirms that observing other customers’ behavior or feeling pressure from a group can heighten the urgency to make a purchase quickly. The desire for social proof often captures customers’ immediate attention in the digital environment, prompting them to seek out information and products quickly. This finding is significant because prior scientific literature primarily linked purchase urgency to factors like scarcity and time constraints, with less emphasis on the influence of group pressure. Despite a somewhat weaker relationship, still observing others’ behavior strongly amplifies interest and social comparison, serving as a powerful catalyst for product evaluation and long-term brand trust.

Extending these findings, we further explore how urgency translates into FoMO. The second strongest impact identified in our study is the urgency associated with FoMO. A sense of urgency significantly contributes to the development of two forms of FoMO: socially driven FoMO and missed opportunities-related FoMO. When consumers feel time pressure, their ability to make informed decisions diminishes, yet the feeling that they must act immediately grows stronger. This leads to intense emotional reactions, which, in turn, explain the marked increase in SD-FOMO in urgent situations. Under time constraints or limited availability, the fear of delay intensifies, especially when they are influenced by social signals, such as others’ purchases, peer recommendations, or current trends (general and induced on social networks). This fear arises from the concern that they will miss out on a product compared to other members of their social group, forcing them to settle for less popular alternatives. Additionally, acting quickly allows customers to present themselves in a positive light by seizing opportunities before others do.

The impact of urgency on opportunities-related FOMO is even stronger, as activities that create a sense of urgency trigger feelings of potential loss. When products are labeled as “last pieces” or when countdowns for offers are presented, consumers may perceive greater benefits and consider lower monetary or non-monetary costs, driven by the fear of missing out. Psychologically, urgency activates mechanisms related to loss aversion, meaning that the emotional weight of losing something (such as a purchase, discount, or product) is greater than the equivalent gain from acquiring it. Consequently, individuals can develop a strong fear of losing the opportunity (Nguyen et al., 2025; A. S. Gupta & Mukherjee, 2022). These factors illustrate why urgency not only aids in decision-making but also serves as a fundamental trigger for FoMO. Even if consumers cannot rationally conclude that an opportunity will indeed be lost, the perception of urgency often leads them to replace rational judgments with emotional responses, such as fear.

These direct effects are further clarified by the mediation analysis results. They indicate that the feeling of scarcity indirectly influences two forms of FoMO through urgency. This suggests that the perceived time pressure is a key mechanism in the development of FoMO. Our findings expand on the evidence provided by X. Zhang and Rosli (2025), which established a direct link between scarcity and FoMO. We propose that there is also an indirect relationship through the feeling of urgency. This aligns with Przybylski et al.’s (2013) observation that FoMO often arises from the perception that significant opportunities are time-limited and quickly disappearing. In this context, urgency can be understood as a trigger for the cognitive-emotional response that leads to FOMO. Additionally, our results show that the perception of restricted freedom of choice, such as time limitations, combined with feelings of scarcity, enhances an individual’s feelings and potentially motivation to take immediate action. This mediation effect should be, however, interpreted with some caution because the high HTMT value between UE and SE indicates potential limitations in discriminant validity between those two constructs. As a result, part of the observed mediation effect may reflect their shared underlying perception of limited opportunity rather than a fully sequential psychological process.

In contrast, the absence of a mediating effect in social proof suggests that it does not act on FOMO via perceived urgency. Past research has highlighted the influence of social proof on the fear of being late (Przybylski et al., 2013; Mahmud et al., 2023) and on behavior (Cialdini, 2008), but primarily through normative and informative social impact rather than through the temporal dimension. Social proof thus likely amplifies FoMO through mechanisms such as social comparison, the need for belonging, and perceptions of social norms. In this context, social proof could influence FoMO primarily through the perception that “others are cooperating” or “others are already enjoying the experience”, rather than through a sense of urgency. Other boundary conditions may be considered when studying this relationship. Since social proof primarily conveys information about a product’s popularity and social validation (e.g., Childs & Jin, 2020), this factor is also important for urgency perception, but we did not take it into account in this study.

A related and potentially interesting question is under what conditions social proof would actually lead to higher FoMO through urgency. Consequently, future research should also incorporate potential moderator variables to test boundary conditions. Factors such as personality traits, the social visibility of consumption, customer involvement, product popularity, and trends could be incorporated into models examining the influence of social proof on FoMO. Also, an experimental study design in which only the experimental group of respondents is exposed to marketing stimuli featuring elements of social proof could reveal potential differences. In a similar way, we could examine situations in which urgency may fail to influence FoMO.

Overall, the findings suggest that FoMO should be understood as a multi-dimensional construct driven by distinct psychological mechanisms, rather than as a single unified response to marketing stimuli.

Also, we show that urgency can act as a crucial mechanism that transforms external marketing stimuli into internal emotional responses. Scarcity reflects constraints in availability, and social proof signals social validation. Urgency appears to be the factor that translates these cues into immediate behavioral pressure. This position of urgency should not be viewed merely as a parallel construct, but rather as a key driver within the consumer decision-making process. While urgency-based cues can accelerate immediate actions, social proof provides the essential foundational validation that aligns a product with societal needs. This suggests that while urgency triggers the final click, social proof substantiates the product’s value and drives sustainable purchase intent.

Importantly, the results highlight that social proof and urgency represent distinct mechanisms that activate FoMO. While urgency operates through time pressure and perceived opportunity loss, social proof functions through social comparison and the need for belonging. Notably, urgency was also found to influence socially driven FOMO. The result can be explained by the fact that urgency and social proof act as distinct psychological triggers of FOMO rather than as sequential processes. Urgency directly activates the fear of missing an opportunity by creating time pressure and a sense that the opportunity will soon disappear, consistent with the principle of loss aversion. In contrast, social proof operates through social mechanisms such as comparison with others and the need for belonging, as explained by social comparison theory. Thus, an individual experiences FOMO because they see others participating or buying, not because they perceive a time limit. Since social proof itself does not inherently signal that an opportunity is time-limited, it does not significantly increase the sense of urgency, and therefore does not indirectly affect FOMO through this pathway.

### 6.1. Theoretical Implications

The results of this research make contributions to the field of the Stimulus–Organism–Response (S-O-R) model. It provides insights into the Stimulus–Organism–Response (S-O-R) model, enhancing our understanding of how various stimuli influence behavioral responses. The scarcity effect and social proof are identified as stimuli, while the urgency effect represents the organism’s internal psychological state. FoMO, which includes both SD-FoMO and MO-FoMO, reflects the response. The scarcity effect influences FoMO indirectly through the sense of urgency, confirming that urgency serves as a mediating mechanism. In contrast, although social proof exhibits a statistically significant direct impact on behavioral responses, it does not operate on FoMO through the same mediating factor. This challenges the theory by demonstrating that an organism’s responses are not straightforward. Instead, these responses are influenced by various psychological processes that can vary depending on the type of stimuli presented or the specific boundary conditions under which the theory holds true. This finding suggests that the organism component of the S-O-R model may be better understood as multiple distinct psychological mechanisms rather than a single unified process, as different types of stimuli appear to activate distinct internal responses.

Additionally, in the context of commodity theory, the results help explain why perceived scarcity affects value perception. The findings show that scarcity not only acts directly but primarily by increasing the sense of urgency, which activates perceptions of potential loss and, consequently, leads to FoMO. In doing so, the research shows why urgency is an important psychological mechanism through which scarcity can influence consumer experience and decision-making.

Interpreting the results through the lens of cognitive dissonance theory helps us better understand how individuals manage psychological tension. A sense of urgency creates conflict between the desire to make informed decisions and the pressure to act quickly, leading to cognitive dissonance. In this context, FoMO can be seen as the emotional response to this tension, stemming from the belief that an opportunity will be lost if one does not act swiftly. Individuals often alleviate this dissonance by rationalizing their decisions or engaging in impulsive behavior. Since social proof does not involve a sense of urgency, the findings suggest that it likely triggers a different type of dissonance related to social comparison and normative expectations, rather than the pressure of time constraints.

The findings of this study indicate that FoMO should be understood as a multidimensional construct, as it is activated through different psychological pathways—specifically, a time-pressure-driven mechanism associated with urgency and a social comparison mechanism associated with social proof—rather than as a single uniform response.

### 6.2. Managerial Implications

The findings of this study highlight the importance of aligning marketing tactics with specific decision-making objectives. Scarcity and urgency emerge as the most effective drivers of immediate purchase behavior, while social proof plays a more limited role in accelerating decision-making.

Given the strong effect of scarcity on urgency, managers should prioritize scarcity-based strategies to create time pressure and stimulate faster decisions. When customers are informed by the media about the scarcity of certain products, suppliers can effectively promote a sense of urgency through advertising or sales promotions. Given our findings and the role of urgency, it makes sense to highlight both scarcity (the limited availability of products in terms of time and quantity) and urgency simultaneously. To emphasize scarcity, companies can use techniques such as showcasing limited quantities or exclusive offers, or creating the impression of high demand. In terms of urgency, strategies may include setting deadlines, using countdown timers, indicating potential price increases, offering time-sensitive bonuses, and providing real-time notifications.

Importantly, the results show that urgency is a key mechanism driving both dimensions of FoMO. This suggests that marketing strategies designed to emphasize time pressure—such as “last chance” messaging or expiring promotions—are particularly effective when the objective is to prevent hesitation and trigger immediate action.

In contrast, social proof appears to play a different role in the decision-making process. The findings show that social proof has a relatively weak effect on urgency and does not indirectly influence FoMO through this pathway. This indicates that its primary function lies in shaping product evaluation, increasing perceived desirability, and building trust, rather than in prompting immediate purchase decisions.

As a result, managers should use social proof strategically in earlier stages of the consumer journey, where the goal is to increase interest, product attractiveness, and perceived value, while relying on scarcity and urgency cues in later stages to convert interest into action. This is supported by the findings, which show that social proof has a relatively weak effect on urgency and no significant indirect effect on FoMO through urgency, indicating that its role is more aligned with shaping product evaluation and perceived desirability than with triggering immediate action.

## 7. Limitations and Future Research

Some limitations must be acknowledged when interpreting the results of our research. First, the use of self-reported data collected with a single measurement instrument at a single point in time can introduce common method bias, leading to overestimation of relationships between constructs or spurious correlations (MacKenzie & Podsakoff, 2012; Podsakoff et al., 2024), since the Harman single-factor test result (46.86%) is close to the 50% ceiling. Future research should include multiple data sources or experimental approaches to reduce this impact.

The second limitation concerns the relationship between perceived scarcity and urgency. The HTMT value (0.908) exceeded the recommended threshold, indicating substantial overlap between them. This finding suggests that respondents may not always perceive scarcity and urgency as fully separate constructs, but rather as closely related manifestations of a broader “limited opportunity” perception. Consequently, the mediation effect proposed in H4 should be interpreted with some caution. While the results are consistent with the theoretical argument that scarcity increases FoMO through heightened urgency, part of the observed mediation effect may also reflect a shared cognitive appraisal of limited opportunity activated by both cues. In this sense, the mediation may capture not only two sequential psychological processes but also a broader underlying perception that combines elements of scarcity and urgency. Future research should further disentangle these mechanisms by employing experimental designs that manipulate scarcity and urgency independently, by developing more distinct measurement scales, or by examining whether both constructs operate as dimensions of a higher-order limited-opportunity construct.

Third, an additional limitation concerns the dimensionality of the FoMO construct. Although the two-factor solution demonstrated superior model fit compared with the original one-factor specification, this structure emerged during the measurement refinement process and was not hypothesized a priori. Therefore, the distinction between social-driven FoMO and missed-opportunities FoMO should be regarded as exploratory. Future research should replicate and validate this dimensional structure using independent samples and dedicated psychometric procedures.

Fourth, the model could include multiple relevant mediators, particularly in the context of social proof relationships, where the indirect influence through urgency has not been confirmed. Potentially, additional constructs, such as social comparisons or perceived social norms, would provide a more comprehensive explanation of the mechanisms at play. Moreover, the research does not account for moderating factors, such as individual differences (e.g., impulsivity, inclusivity, demographic factors, psychographic factors) or situational contexts, which can influence the strength of the identified associations.

Fifth, a cross-sectional research design limits causal inference. Future time-lagged study design or experimental studies could more reliably verify the direction and dynamics of the relationships between constructs. Specifically, measuring social proof and scarcity, as well as urgency and FOMO at three different time intervals, would reduce concerns about common method bias while also providing stronger evidence for the hypothesized causal chain. This design would further help address the observed construct overlap between SE and UE by allowing their effects to unfold over time. It could potentially also improve discriminant validity and yield more stable mediation estimates.

An experimental design could also be used to examine the causal effects of SE, SP, and UE on FOMO. In such a setup, participants in experimental conditions would be exposed to manipulated scenarios featuring high scarcity (e.g., limited product availability), strong social proof (e.g., high popularity or positive user behavior), or urgency (e.g., urgent purchase deadlines), while a control group would receive neutral scenarios without these cues. Comparing the two groups would allow for a more rigorous assessment of the causal impacts.

Finally, the generalizability of the results is limited by the less representative sample and the specific context of the research on online purchases, so it would make sense to test the model in other cultural and market settings.

## Figures and Tables

**Table 1:** Sample characteristics.

Gender	Frequency	Percent
Other	1	0.8
Male	47	38.8
Female	73	60.3
Total	121	100
Age		
18–24	29	24
25–34	56	46.3
35–44	23	19
45–54	7	5.8
55–64	3	2.5
Do not want to answer	3	2.5
Total	121	100
Education status		
Primary school	4	3.3
Secondary school	43	35.5
Higher vocational/professional education	25	2.7
University education (undergraduate)	32	26.4
Master’s degree	13	1.7
Doctoral degree	2	1.7
Do not want to answer	2	1.6
Total	121	100

**Table 2:** Latent constructs and Items, Means, Standard Deviations, CFA Loadings, AVE, and CR for all included latent variables.

Latent Constructs and Items	Means	Std. Deviations	Lambdas	CR	AVE
Scarcity effect (SE)					
If a product is part of an exclusive limited-quantity series, I am more likely to purchase it.	3.44	1.736	0.792	0.881	0.713
If a website indicates that only a few items are left in stock, I am more likely to purchase the product quickly.	3.50	1.669	0.833		
When I see that a product has limited stock availability, I am more likely to purchase the product quickly.	3.52	1.728	0.903		
Urgency effect (UE)					
When a countdown timer is displayed on a website, I am more likely to purchase the product quickly.	3.40	1.832	0.800	0.874	0.700
Flash sales (short-term discounts) make me more likely to purchase products that I would not otherwise purchase.	3.53	1.703	0.793		
When I see labels such as “limited-time offer” or “ending soon,” I am more likely to purchase the product.	3.48	1.718	0.911		
Social proof (SP)					
Reviews and ratings from other customers influence my decision to purchase a product.	4.74	1.627	0.800	0.872	0.630
When choosing products online, I often rely on recommendations from others (e.g., friends, family, acquaintances).	5.02	1.514	0.810		
Negative reviews or bad experiences of other customers often discourage me from purchasing a product.	5.31	1.565	0.731		
When I see that a product has many positive reviews, I am more likely to purchase it.	4.87	1.511	0.831		
Social driven FOMO (SD-FOMO)					
I fear that my friends and other consumers are purchasing better products online than I am.	2.93	1.595	0.602	0.882	0.657
I worry that I might miss out if I do not purchase products that my friends have purchased.	2.95	1.875	0.976		
When I see others purchasing a popular product, I feel a need to purchase it quickly as well.	2.97	1.717	0.772		
It is important to me to purchase the same products as my friends.	2.91	2.025	0.847		
Missed opportunities FOMO (MO-FOMO)					
It bothers me when I miss a one-time opportunity to purchase a product.	3.88	1.763	0.804	0.839	0.723
It bothers me when a popular product I want to buy online is sold out.	4.37	1.785	0.894		

**Table 3:** Correlations between latent variables and Square Roots of AVE.

Title 1	1.	2.	3.	4.	5.
**1. SE**	**0.844**				
**2. UE**	0.882	**0.836**			
**3. SP**	0.593	0.613	**0.794**		
**4. SD-FOMO**	0.491	0.607	0.435	**0.811**	
**5. MO-FOMO**	0.771	0.733	0.609	0.498	**0.850**

All correlations were significant at *p* < 0.001. Bolded values indicate the Square Roots of Ave. Abbreviations: Scarcity effect—SE; Urgency effect—UE; Social proof—SP; Social-driven FOMO—SD-FOMO; Missed-opportunities FOMO—MO-FOMO.

**Table 4:** Discriminant validity of Heterotrait-Monotrait Ratio of Correlations (HTMT).

Title 1	SE	UE	SP	SD-FOMO
**UE**	0.908			
**SP**	0.612	0.591		
**SD-FOMO**	0.575	0.654	0.498	
**MO-FOMO**	0.789	0.760	0.612	0.566

Abbreviations: Scarcity effect—SE; Urgency effect—UE; Social proof—SP; Social-driven FOMO—SD-FOMO; Missed-opportunities FOMO—MO-FOMO.

**Table 5:** Hypotheses and direct and indirect relationships between latent variables.

Direct and Indirect Impacts	Estimates	Significance
Direct impacts		
H1: SE → UE H2: SP → UE H3a: UE → SD-FOMO	0.787	*p* < 0.001
	0.182	*p* < 0.05
	0.557	*p* < 0.001
H3b: UE → MO-FOMO	0.795	*p* < 0.001
Indirect impacts		
H4a: SE → SD-FOMO	0.438	*p* < 0.001 95% CI = [0.282; 0.614] SE = 0.086
H4b: SE → MO-FOMO	0.625	*p* < 0.001 95% CI = [0.424; 0.869] S.E. = 0.113
H5a: SP → SD-FOMO	0.101	n.s. 95% CI = [−0.034; 0.237] S.E. = 0.071
H5b: SP → MO-FOMO	0.145	n.s. 95% CI = [−0.047; 0.328] S.E. = 0.098
Control variables		
Gender → UE; SD-FOMO; MO-FOMO	0.007; 0.011; −0.027	All n.s.
Age → UE, SD-FOMO, MO-FOMO	−0.013; −0.157; 0.015	All n.s.
Education → UE, SD-FOMO, MO-FOMO	−0.050; 0.068; −0.180	All n.s.
Fit indices: χ^2^ (132) = 233.97; TLI = 0.904; CFI = 0.926; IFI = 0.928; RMSEA = 0.080		

Abbreviations: Scarcity effect—SE; Urgency effect—UE; Social proof—SP; Social-driven FOMO—SD-FOMO; Missed-opportunities FOMO—MO-FOMO.

## Data Availability

The original data presented in the study are openly available in Zenodo repository at https://doi.org/10.5281/zenodo.19595283.
